# Ethics and metaphysics of cadaveric organ conscription for transplantation

**DOI:** 10.1007/s40592-025-00268-9

**Published:** 2025-11-13

**Authors:** Bjørn Hol

**Affiliations:** Centre for Medical Ethics, Institute of Health and Society, the Medical Faculty, University of Oslo, Oslo, Norway

## Abstract

There is a shortage of organs for transplantation resulting in long waiting lists, and many die while waiting for a new organ. Different policies are discussed for solving this problem, but the most effective, namely the conscription of cadaveric organs, the routine taking of organs from dead bodies, is considered too controversial and is therefore not implemented anywhere.I discuss the reasons for and against conscription; the former are mainly based on utilitarian arguments, while the latter are mainly based on the interests and rights of the deceased and his family. (He, his, or him, is understood to mean he/him/his or she/her/hers throughout the article.) .This article investigates whether the deceased has any interests and rights. I argue that dead bodies are mere things and thus without interests or rights. I also discuss whether the interests of the family should override the needs of patients who are on waiting lists for organs.Using arguments based on Epicurean philosophy, and supported by contemporary scholars, I conclude that dead bodies do not have interests and rights and that the possible interests and rights of the dead’s family cannot be more important than saving the lives of the people on waiting lists for organs.Thus, I argue that conscription of cadaveric organs for transplantation can be defended and should be implemented as a normal hospital practice.

## Introduction

Organ transplantation is a successful treatment for many serious health problems. However, only a few organs can be taken from live donors, and live donors are too few to meet the needs of people on waiting lists for organs. Many more organs can be taken from dead bodies. But using cadaveric organs needs consent, either from the antemortem person, from the family or from both, depending on the legal regulation in the country. And in some cases, consent is denied, and usable organs are instead buried or burnt.

There are several issues in the bioethical debate on organ transplantation using cadaveric organs (Veatch and Ross 2015). Those relate to questions of procurement, and of allocation. One question is about the definition of death. In this article I do not comment on this, just presuppose that the donor is dead. Questions related to the removal of cadaveric organs, and justification for procuring organs are discussed. Principles of fair distribution of organs is not the focus of this paper. I do, however, mention a potential problem with “free-riders”, persons requiring organs if in such need but deny being potential donors.

There seems to be a gap in the scholarly debate on the relevance of the cadaver’s lack of moral status for the legal regulation of transplantation activities. Therefore, I hope that a discussion of the transformation from a human being into a mere thing through death,^1^ which I argue is also a transformation from intrinsic to extrinsic value, will show that conscripting cadaveric organs should be adopted as a general policy.

Thus, this article focuses on questions of procurement and is motivated by the shortage of organs for transplantations.^2^ The supply of organs can be increased in several ways (e.g., by establishing a commercial market for organs from living and/or dead donors, a strict opt-out system, or conscripting^3^ cadaveric^4^ organs). My focus in this paper is on this last possibility.

I use the situation in Norway as a case, but the discussion is relevant for most countries with transplantation activities. However, there are relevant differences between countries that make direct comparisons difficult.

I do not focus on the different theories on posthumous harm. I do, however, explain the most common arguments for and against posthumous harm, which is understood as the thwarting of posthumous interests. The wrongness of thwarting the antemortem person’s posthumous interests is one commonly used argument against disrespecting the antemortem person’s wish and will.

The aim of this article is to defend the conscription of usable cadaveric organs for transplantation, which provides a direct counterargument to discussions in the preliminary documents (the white papers) and the resulting paragraphs in the Norwegian law on organ transplantation (“Lov om donasjon og transplantasjon av organ, celler og vev (transplantasjonslova),” n.d.) (*Når døden tjener livet. Et forslag til nye lover om transplantasjon, obduksjon og avgivelse av lik.*, 2011), (*Prop.38 L *(2014–2015)* Transplantasjonslov og obduksjonslov*, 2014), (“Lov om donasjon og transplantasjon av organ, celler og vev (transplantasjonslova),” n.d.). The white papers mention the possibility of introducing conscription in Norway but discard this possibility as too controversial.

In this paper, I use analogies both descriptively and normatively to make my arguments more convincing.^6^

I proceed as follows. First, I present relevant Norwegian legal provisions on the use of cadaveric organs for transplantation. Then, I discuss general questions on why organ transplantation, and especially organ conscription for transplantation, is important and how and why it can be justified through theories of distributive justice and other ethical theories. These arguments are primarily from the perspectives of potential organ recipients. There is also an often-overlooked argument based on the “free-rider” problem, which is solved through conscription. I discuss counterarguments based on the antemortem person and their family’s perspectives. In addition, I present an Epicurean argument for the thingness of the corpse and briefly mention the contemporary debate on the status of the dead. Since some arguments against the conscription of cadaveric organs are based on an understanding of thwarting the interests of the dead, I briefly present common arguments in the debate on posthumous harm. I also present a problem about ownership and the transfer of ownership of cadaveric organs and propose a solution, after which I conclude the paper.

1. **Norwegian legal provisions on the use of cadaveric organs for transplantation** (*Act relating to donation and transplantation of organs*,* cells and tissues (Transplantation Act)*, 2015, §§ 10–15) (*Act relating to donation and transplantation of organs*,* cells and tissues (Transplantation Act)*, 2015).

If the dead gave consent before death, organs can be taken for transplantation and family/next of kin cannot decline the donation.

Lacking such consent, organs can be taken, but not against the will of next of kin, and not if there are no next of kin, or contact with them has not been possible.

If the deceased is known to have objected to donation, organs cannot be taken, even with the family’s consent.

Thus, the Norwegian provisions represent both an opt-out and opt-in policy (if there is no family) – the last having the least persuasive argumentation, namely that if at a later time, after the taking of organs, a family turns up, they can be distressed if organs have been taken from their deceased family member without their knowledge.

As a result, the Transplantation Act occupies a middle position. If the deceased’s will is known, it is to be respected. If it is unknown, organs can be taken as long as the family does not object. However, if the deceased’s will is unknown and there is no family or contact with the family is impossible, then their organs cannot be taken.

## Distributive justice: reasons for cadaveric organ transplantation

The Norwegian healthcare system typically prioritizes resources based on health benefits, resource use, and severity (i.e., absolute QALY shortfall; *Det viktigste først Prinsipper for prioritering i den kommunale helse- og omsorgstjenesten og for offentlig finansierte tannhelsetjenester*, n.d.). The transplantation of cadaveric organs meets all of these principles of priority setting and should therefore be uncontroversially and automatically on the top when it comes to the use of healthcare resources, if one accepts my argumentation in this article.

The distribution of benefits and burdens across members of society does not normally focus on the redistribution of organs. However, some scholars have argued (Fabre 2003, 2006a; Erin and Harris 2003; Harris 2013) that, if there are regulations for the redistribution of material goods, why should there not also be regulations for the redistribution of organs from those who can donate them to those in need? Since my focus is on cadaveric organs, I only discuss which principles could justify taking organs from the deceased and transplanting them in those who need new organs. Examples of relevant principles are as follows (Lamont and Favor 2017; Rosoff 2018).

First, strict egalitarianism is a principle of equality. Since some people have better health than others and health is important for well-being, most would agree that a society in which one receives health service when one is in need is preferable to one in which this is not the case. Although health services do not have the ability to secure the same good health for all, most people would presumably accept this as an ambition for their health service within certain economic and other restrictions. Therefore, when organs can be used to improve the health of those in need without making anyone else’s health worse, this seems to be a net good.

Second, welfare-based principles, like utilitarianism, should be a reason to take organs from those who no longer need them and give them to those who do. The benefit would be great for receivers,^7^ and no one’s life would be any worse (except maybe those of the deceased’s family if organs are taken against their will). Therefore, transplantation results in more utility.

Third, it is commonly accepted that we all have a duty to rescue if this can be done without risk to oneself. The taking of organs from the dead is “risk-free”^8^ for the dead and is of crucial importance for the receiver.

Fourth, principles of distributive justice seem to be of relevance since people who are on waiting lists for organs are among the worst off in society. Receiving a much-needed organ would increase their well-being and expected longevity. Furthermore, as organs would be more plentiful if conscription of all usable organs where the routine, restrictions on who receives an organ may be liberalized and help those who would otherwise be exempted from transplantation.

The above principles, which are based on weighing the benefits and burdens of the donors and the recipients of organs, are not the only kind that can be used for conscription. A principle of fairness also seems to be relevant. In this case, it is about reciprocity. Therefore, I add the following principle:

Fifth, a principle of reciprocity seems to be fair in our case of using cadaveric organs for transplantation. When some people refuse to be donors, it seems fair to use a principle of reciprocity in such cases. This would imply that they would not be potential recipients of organs, or be prioritized second, favoring those willing to also be donors. However, conscription would remove this problem since everyone would be potential donors (Fleck 2022). The same principle should also be used for vetoing family member(s) which then would also not be potential recipients of organs or be on a lower priority for organs. I have no statistics on how many there could possibly be, but principally, this would also procure more organs for those on waiting lists (Alnaes 2012).

## Why conscription?

As previously mentioned, there are several ways to increase the supply of cadaveric organs for transplantation. One could try to make more people agree to be donors by formally registering for and carrying donor cards (e.g., on their phones), which is typically called an opt-in system. Alternatively, there can be a system in which people must formally reject becoming a donor; otherwise, they are understood to agree to organ donation. This is typically called an opt-out system. A third option is to have a system like in New Zealand, where people must choose whether or not to become donors before obtaining a driver’s license. In addition, it is possible to invest in a follow-up system in which the families of the newly dead are visited by healthcare personnel to inform them about the use of cadaveric organs for transplantation, which has been very successful in Spain (Gil-Díaz 2009). Finally, it is possible to establish a marketplace for organs in which people could sell their own organs (for delivery after death) or families could sell the organs of the newly dead.

However, none of these systems is 100% effective. The most effective way to procure more organs would be to simply make the taking of suitable organs from the newly dead routine (Sterri et al. 2022). All contemporary regulations that rely on consent from the antemortem person or his family (or both) result in organs that could have been used to help people on waiting lists to live better and longer lives being buried or burnt instead.

Many scholars support a policy of conscripting cadaveric organs. Arguments for such a policy include the effectiveness of securing more organs; the elimination of stress on healthcare personnel when asking for consent and families when giving consent; saving time when waiting for approval, time that may reduce the quality of organs; the equitable sharing of burdens and benefits; and the moral duty to help when there is little or no risk or burden on the helper (Spital and Erin 2002; Spital 2003, 2005a, b; Fabre 2006a; Hester 2006; Howard 2006). There are additional arguments for conscription using analogies such as taxation, military drafts in wartime, mandatory vaccination, and similarity to forensic autopsies. People accept that autopsies are performed when there is suspicion of criminal acts or possible public health threats (Spital and Taylor 2007, 2008; Delaney and Hershenov 2009; Taylor 2014; Rosoff 2018). Furthermore, there are strict rules and regulations related to the disposal of dead bodies, which are not at the family’s discretion, and which are normally accepted by the family.

In 1985, John Harris (Harris 2001) argued in favor of moving from an opt-in system to an opt-out system in which people should be motivated to see death as an opportunity to help others; thus, opting out would be seen as supreme disrespect. He did not advocate for conscription because it could be seen as a tax, which could lead to resentment. Instead, he encouraged people to donate organs as a last opportunity to exercise virtue. However, if this was not enough to procure the needed organs, he recommended a system of ordering organ procurement. In 2013, he argued,

…considerations for the welfare and interests of the dead and the philosophical attention given to them are self-indulgent nonsense at best, and at worst a crime against humanity. The real issues are the extent of the harm that might be caused by not using tissue, organs, cells, DNA, and other biomaterials from the dead, and the extent of the good that using such biomaterials might achieve (Harris 2013, p. 213).

Analogies can often help to clear the mind and resolve questions. With regard to reusing cadaveric organs, several analogies are possible. For example, in the affluent Norwegian society, people often change their wardrobes due to changes in fashion or because they simply want something new. Their discarded but fully usable clothing is given to organizations that sell used clothes. Thus, an old coat can be reused by someone else (Emson 2003). There is a market for used cars. They may be fully operational, but the owner wants something new. In a future point, the car can also be used for spare parts. The reuse of things for the benefit of the receiver is a well-known practice. Thus, the use of cadaveric organs (spare parts or waste) to help others live longer and better lives (repair) is akin to using taxpayer money (conscription) to finance healthcare in general. It simply seems like the rational thing to do, like the reuse of things that have lost their value for the donor but can have value for a receiver. Similarly, cadaveric organs have no value for the corpse but immense value for the receiver.

There seem to be many good arguments for conscription. But the permissible and even recommended donation of organs can become an unlawful act if it is performed against the will of the deceased or his family (Steiner 2002). This seems to presuppose that the deceased and his family have interests and rights that should be respected.

## Counterarguments based on the family’s perspective

My arguments for conscription are supported by arguments for the nonvalue of death^9^ and the transformation of a human being into a mere thing that happens at death, which also makes the intrinsic value of a human being change to the extrinsic value of the corpse for others (on waiting lists for organs). However, there are three reasons why the corpse may be held to be more than a mere thing.

First, corpses are legally protected by punishable provisions. One can neither do to a human corpse what one can do to mere things, nor what society deems to be morally wrong.

Second, a strong intuition guides people to treat a newly dead family member with respect. Moreover, our intuitions are not only restricted to family members. Our feelings and intuitions toward dead bodies are not the same as our attitudes toward other things. Family members who see their next of kin seemingly breathing, with a beating heart, and warm to the touch because connected to a respirator, may find it difficult to accept that the body is a mere thing and that its organs should be used to help people on waiting lists.

Some intuitions and feelings are deep-rooted and maybe irrational, but they are not easily eradicated. Having some respect for dead bodies is one such feeling. Therefore, there are limits to what most people would accept in relation to the bodies of their next of kin. However, it is acceptable to burn or to bury them, probably because this is part of our culture and has been for a long time. Feinberg (1985) discussed people’s emotions toward dead bodies and the use of cadaveric organs for transplantation and argued that, although a total disrespect for dead bodies could weaken important human sentiments and would therefore be harmful, the use of cadaveric organs is not a practice that falls into this category. Moreover, he criticized William May’s (May 1973) argument that respect for a dead body as a symbol of the living human should take precedence over saving lives.

It seems problematic to accept a system in which dead bodies are connected to the necessary apparatus to keep their blood circulating, held in a sort of warehouse, and used as “spare parts” when needed; as surrogates, such that embryos could be placed in a woman’s uterus and gestated to term (Smajdor 2023); to test new medicines; or to train medical students. I suppose many balk at such an idea, and it is utterly unthinkable that any politician could propose it. Willard Gaylin, as explained by Feinberg, makes a convincing case for the establishment of what he called bioemporiums in which brain-dead bodies are stored for later use. However, although his case seems convincing, he himself was not convinced. Feinberg, discussing Gaylin’s case, although accepting that one may say the case is repugnant, thought that the obvious humanitarian benefits could outweigh repugnance.

Feinberg argued that the benefits of using cadaveric organs outweigh the possible burdens of negative feelings over the use of dead bodies to help people live longer and better lives. Moreover, he believed that it is possible to maintain our human sentiments in an effective humanitarian system in which cadaveric organs are used to help people on waiting lists.

Third, although rituals surrounding the disposal of dead bodies differ around the world, these rituals are important, which demonstrates the strong feelings connected to the dead body, representing the former living person. For example, in Ghana, people believe in the concept of ancestry and that the dead continue to exist in other realms. Because the dead still interact with the living and have some interests, the latter must be weighed against the needs of the living. Thus, there is acceptance of possible posthumous harm in Ghana (Banyubala 2016).^10^

The idea of ancestry with interests and rights seems similar to Daniel Sperling’s proposal that we should adopt a concept that he called the Human Subject (Sperling 2008), which is understood to be nonmaterial, but to have interests and rights beyond the time of the actual subject’s lifetime. This construction may help resolve some of the difficult questions related to posthumous harm, ownership of the dead body, the taking of cadaveric organs without consent, and respect for wills and wishes made by the antemortem person. The concept of a Human Subject is therefore attractive because it seems to capture the notion of respect for the dead in many ways, as well as respect for the dead body, both of which are in accordance with our intuitions. However, as I argue that, if necessary, organs should be conscripted regardless of the will and wish of the dead (or the family), I must depend on my arguments for the interestless and rightless corpse based on the concept of its mere thingness, in accordance with Epicurean philosophy. This also implies that the routine taking of cadaveric organs can be said to be “risk-free” for the dead since any form of interests, rights, or posthumous harm is denied.

Our feelings toward the dead seem to contradict the claim that the dead body is a mere thing.^11^ As Ellen Stroud said, “And this is the central puzzle of the law of the dead: The human corpse is a thing, a material object – a messy, maybe dangerous, perhaps valuable, often useful, and always tangible thing – and the law has much to say about such things. However, the dead human body is also something very different: It is also my father, my friend, perhaps my child, and someday, me. For even the most secular among us, a dead human body is at the least a very peculiar and particular kind of thing” (Stroud 2018, p. 227).

Can our feelings toward the dead body transform it from a mere thing to something else?

One could argue that feelings have the capacity to change the properties of a thing, such as when one discovers that a presumed copy of an original painting is, in fact, the original, which makes it more valuable. However, again, this is not about the properties of the painting (as it has not changed) but rather evaluation. The evaluation of the painting is relational and depends on what people think about its value, not only the properties of the painting itself.

Against the strong feelings of family members who gather around their newly dead relative, it would be disrespectful to claim that the corpse in the coffin is merely a thing. Moreover, it would certainly cause horror if someone smashed a hammer into the face of the dead. However, using parts of the dead for transplantation should not diminish or destroy the emotions experienced by the family. If anything, the thought that the dead has helped other people achieve better and longer lives could alleviate their sorrow, as is also reported by Alnæs (Alnæs 2001).

Can these consequentialist arguments for the taking of organs be convincing against the family’s feelings? They certainly can. On the one hand, we have the feelings of family and friends; on the other, we have dying patients who are in need of organs. We know that most people in Norway (70–80% of the population, according to statistics from the Foundation for organ donation) would agree to donate their relative’s organs. It is likely that some people may feel this to be problematic. However, that a majority consents to the taking of organs from their dead relative, may show that they accept that saving the lives of people on waiting lists trumps any distress that they may feel.

In most countries, the family can veto the retrieval of cadaveric organs for transplantation (but not in Norway if the deceased has agreed to be a donor). Thus, it is important to understand why the family has such authority. The arguments can be divided into two main groups: the authority gained from the dead and the family’s intrinsic authority. The first presupposes that the family knows what the dead wanted and can therefore act on his behalf. The second presupposes that the family has its own authority, regardless of what the dead may have wanted. The first group of arguments assumes that what the dead wanted matters and that he had an interest in the matter—an interest that survived death and that the family now represents. The second group of arguments assumes that the family has interests beyond representing the interests of their dead relative. Furthermore, both groups of arguments assume that the interests in question outweigh other, possibly relevant interests (e.g., the interests of potential organ recipients).

The first alternative, namely that the family represents the dead and, in this capacity, knows his wishes and ensures that they are followed, is similar to a discussion on the deceased’s own interests and rights, which I discuss in ch. 6. However, there are two complications that I do not develop in this paper.

First, I do not discuss whether the family is, in fact, able to know what the dead wanted, as this is more of an empirical than a philosophical question. Second, I do not discuss how or why the family gained the authority to represent the dead. This is taken for granted when it comes to all practicalities regarding the disposal of the dead body and the funeral and is of lesser philosophical interest.

Then, the main question is what the family’s own interests are in the cadaveric retrieval of organs. The feelings of the family can be strong, as shown by examples from, for instance, the United Kingdom (Sque et al. 2008) and Norway (Alnæs 2001). As mentioned above, it seems absurd to say that it is better to burn or bury usable organs than to use them to save lives. One explanation that some think this, in Norway, a minority of about 20%, is that in some cases the family, seeing the dead body with a ventilator keeping circulation going, does not accept that the person is dead. They still have hopes that he can be saved. In such cases, the doctor has the important task of informing the family that their relative is dead, but that the ventilator is keeping the heart and breathing artificially continuing. What they are seeing is a dead body, not their next of kin.

Another reason may be that some people falsely believe that the body will be cut up, such that it will not be viewable by the family afterwards. I have heard this in personal conversations with friends; Alnæs also reports this (Alnæs 2001).

Others are distressed by the thought that the dead body is not “complete” when it is buried or cremated.

In the United States, there is a low willingness to donate organs among African Americans for several reasons. These include the belief that the organs are inequitably distributed, distrust in the healthcare system, the fear that a donor card will lead to a premature declaration of death, and religious reasons (Veatch and Ross 2015). Low willingness to donate is a significant problem since the rate of kidney failure is much higher among African Americans than some other ethnic groups.

In any case, one may hold that any distress that the family might experience is much less important than saving lives and improving the well-being of many people on waiting lists. It is contrary to common intuitions that sensibilities are more important that saving lives (Harris 1992).

Nearly all religions support organ donation. However, some minority groups may have severe problems with it. Therefore, if these problems are of a sort that would make their well-being very much lower, it would probably be best to allow for an opt-out for them. However, this could make people balk at accepting such people as “free riders.”^13^ If so, this problem could be solved by not accepting them as potential organ recipients (Loewy 1996) or, perhaps less controversially, giving them second priority when organs are available for transplantation (Peters 1988). 

Some scholars have highlighted another argument against conscription: the loss of the “gift relationship” (Steiner 1994; Perry 1981). The importance of the gift relationship (i.e., the good feeling that one may experience from giving a gift to someone) necessitates both that one gives the thing freely and that the thing is one’s to give.^14^ Moreover, Steiner indicated the necessary premise of “self-ownership” of one’s organs and argued that this is a fundamental human right. Steiner also asserted that our original property rights must include ourselves: “Our bodies must be owner-occupied” (Steiner 1994, p. 232). Therefore, I briefly discuss the problem of ownership and the transfer of ownership in relation to the issue of organ transplantation in Chap. 7.

## The Mere thingness of the corpse

The philosophical debate that is relevant to my arguments concerning the thingness of the corpse centers around the following issues.

What is our essence? What is a necessary property that we have and would not exist without? And what happens at death (i.e., when we die)? Do we still exist after death as a corpse, or do we cease to exist, having been transformed into something different—namely, a mere thing?

I claim that life is our essential property. We are no longer the same entities after death, as also argued by Hershenhov (Hershenov 2005): “I would insist that an organism is *essentially* a living entity” (p. 49), and “Given all the problems that the body poses, I believe that we are warranted in concluding that each of us neither has nor is identical to a body that will continue to exist after our deaths.” (p. 57).

However, this idea is not uncontroversial. Fred Feldman said that, if we claim that a certain property—call it “C”—is the essential property of a person and that he loses this property when he dies and therefore ceases to exist, he would simply deny the claim that C is an essential property and that the person still exists after death as a corpse (Feldman 2000). (I return to Feldman’s arguments below.)

Stephen Wilkinson discussed what it means to say that a body is more than a mere object and argued that its relation to the person whose body it is makes the significant difference between the body as an object and a mere object. It may follow that a dead body, no longer having this relationship with the person it once was, is a mere object, or a mere thing (Wilkinson 2003). Wilkinson’s argument may partly explain our intuitions that cadavers have a lesser value than living persons. However, it does not fully explain why we also hold that dead bodies are more valuable than the coffin, flowers, or candles that surround the coffin. Maybe it is their morphology that makes us feel this way. We identify dead bodies with human beings because they are very similar to human beings. They look like human beings, which makes it difficult to accept that dead bodies are something very different (Kipke 2020). We know this but cannot fully accept it. Moreover, this is probably also why legal provisions regulate how we should treat dead bodies and that most people find it natural to treat them with respect.

Wilkinson also argued that the dead are neither people nor mere things^15^ and that we should respect the dead because of the interests that people may have had while they were alive with regard to what happens to their bodies after death (Wilkinson 2014; see more on this in ch. 6).

However, there is another interesting issue that Wilkinson seems to overlook: the moral status of dead bodies seems to be determined by our thoughts about them. He argued that some people may see dead bodies no differently than furniture, which do not deserve any ethical consideration. Others, like himself, believe that dead bodies deserve respectful treatment. Then, is the moral status of the body an objective or a subjective property? Does what *I* feel about this question determine not only what I think is the moral status of the dead body but also what the moral status of the body *is*—for me? This question must be answered in another paper.

### What is our essence?

The Epicurean position provides two reasons for the acceptability of conscripting cadaveric organs for transplantation. First, it explains why the dead body is a mere thing. Second, it clarifies why the taking of cadaveric organs does not concern the dead, as nothing matters for the dead. Epicurus famously held that death is nothing to us because, when we are, death is not, and when death is, we are not:

Get used to believing that death is nothing to us. For all good and bad consist in sense-experience, and death is the privation of sense-experience […]. For there is nothing fearful in life for one who has grasped that there is nothing fearful in the absence of life […]. So, death, the most frightening of bad things, is nothing to us; since when we exist, death is not yet present, and when death is present, then we do not exist. Therefore, it is relevant neither to the living nor to the dead since it does not affect the former, and the latter do not exist. (Morgan 1992, pp. 417).

This passage presents the main arguments in what is often called the philosophy of death. First, Epicurus, a hedonist, argued that sense experience is a necessary condition for anything to be good or bad. Moreover, when we are dead, we no longer sense anything. He further claimed that death is nonexistence, and that existence is also a necessary condition for anything to be bad. An Epicurean understanding of death implies that the dead cannot be harmed since harm requires an experiencing subject.^16^

Thus, I presuppose the termination thesis (Epicurus was a terminator, in Feldman’s words [Feldman 1992 p. 92]; i.e., death is nonexistence). When one dies, one ceases to exist as a subject and is no longer part of the world, as also argued by Sumner (Sumner 1976). Of course, one may still be part of the world of others (e.g., in their memories and when they take care of the dead body, arrange the funeral, and take care of the material property). Although biology ends, the biography does not (at least not immediately), and one may continue existing in the memories of others.^17^

Therefore, the body is still, for some time, part of the world and can be of use. The former person is no longer a subject but an object, a mere thing. It is not controversial to call the dead body a “thing.” However, what does “mere” add to the “thingness” of the body? According to Peter van Inwagen, thing is a concept that quantifies over all there is, concrete and abstract (Van Inwagen and Zimmerman 2007). It covers living animals such as human beings and all else that exists. However, in my understanding, a mere thing excludes living animals, as the latter have intrinsic value, at least those with a mental life. Therefore, I hold that there is an important distinction between things, which means everything, and mere things, which exclude living beings with intrinsic value.

For Epicurus, a distinct difference between the living person and the dead body was important. His project was to alleviate the fear of death—of the state of being dead. Thus, he argued that the dispersion of atoms that compose both body and soul imply the cessation of existence, experience, and any possibility of harm. Therefore, Epicurus would support an understanding of the corpse as a mere thing without any intrinsic value. As James Warren also indicated, Epicurus would insist on a clear separation of the antemortem person and his postmortem remains (Warren 2002). In support of this view, Lucretius emphasized that, if someone complains about what happens to his corpse (rotting or being eaten by beasts), he has not fully accepted the truth of the Epicurean distinction between the living person and the corpse (Lucretius 1994).

If the corpse is a mere thing, moral theories would concur that it lacks intrinsic moral value. I also claim that it lacks interests and therefore rights, which is also stated in the Official Norwegian Report (White Paper) - *When death serves life. A proposal for new laws on transplantation*,* autopsy and delivery of corpses* (NOU 2011: 21, p. 125), although it also seems to assert that the dead have property rights to the body (*Når døden tjener livet. Et forslag til nye lover om transplantasjon*,* obduksjon og avgivelse av lik.*, 2011). Therefore, the corpse cannot plausibly be harmed in any way.

The Epicurean theory on the nonvalue^18^ of death is supported by many scholars. However, more researchers support the deprivation theory on the badness of death, which holds that death is bad for the deceased because of the future life that he was deprived of. However, this is not problematic for the claim that the corpse is a mere thing. My argument for this thingness is based on the idea that death is a transformation; the former living body is transformed into a corpse, and the corpse is a mere thing. The former living human being no longer exists. Moreover, this may also be supported by deprivationists. For example, there is Thomas Nagel’s first sentence in “Death”: “If death is the unequivocal and permanent end of our existence, the question arises whether it is a bad thing to die” (Nagel 2009, p. 1). Thus, the deprivationists (including Nagel) require the termination thesis for their argument that the loss of life (i.e., the transformation of the body into a mere thing) is the badness of death. The Epicurean position requires the nonexistence of the person for the argument that there is no loser and therefore no loss. Thus, both sides in the debate on the badness of death support the claim that death means that the once-alive person no longer exists because he is transformed into a corpse, which is a mere thing.^19^ However, not everyone (e.g., Feldman, which I discuss below) supports the idea that the corpse is a mere thing.

The deprivationists seem to hold that the dead are somehow harmed or at least have their interests thwarted by losing their counterfactual future life. This seems to provide the dead with some “afterlife.” Deprivationists make assumptions about what the future life of the now dead person could have been. If the life is supposed to have been good, they hold that death was bad; if the life is supposed to have been bad, then death was less bad or even good. Deprivationists would perhaps be more likely to see the conscription of cadaveric organs to be against the antemortem person’s will and as thwarting the interests of the antemortem person (if not the dead), which could be held to be bad. Moreover, this leads to the philosophical debate called posthumous harm, which I discuss in ch. 6.

### What happens to Us when we die?

The answer to the above question seems to be rather obvious to most people: we no longer exist because all our functions and capacities shut down. We are no longer part of the world of the living. Jeff McMahan presented four options for how to view the dead body (McMahan 2002). First, the dead body can be viewed as a new entity that comes into existence through death. Second, one can accept that the corpse has existed all along but is not identical to the antemortem body. Third, the antemortem body is a fundamental entity that encompasses both the living body and the corpse. Finally, one may deny that there is a corpse and that all there is, are cells that are organized to look like a corpse.

Thinking of the dead body seems to be less about ontology and more about semantics. It concerns how we should describe the corpse. We do not lack any necessary facts needed for a correct description. My claim that death transforms the living human being into a mere thing seems to be best captured by McMahan’s first option—namely, that the corpse, which is different from the living person, comes into existence by a transformation caused by dying. But it sounds a bit awkward to put it this way. In normal conversation, I suppose that one could simply say that the person has ceased to exist and is now a corpse since a corpse is what we call a dead body (Olson 2004). Others find it more intuitive to think that the corpse somehow must have existed before death, since nothing new has been created (Shoemaker 1999).

Feldman, who introduced the termination thesis which holds that people cease to exist when they die (Feldman 1992), does not endorse this thesis and argues that a member of the species *Homo sapiens sapiens* is still a member even after death (Feldman 1992, 2000). Feldman’s intuition is that the cadaver remains a member of the species *Homo sapiens sapiens* and that a dead horse is still a horse. I must discuss this a bit further since it is a direct counterargument to the claim that death transforms the human being into a mere thing. Feldman discussed three notions of personality and their implications for the termination thesis: the biological, psychological, and moral conceptions of personality (Bradley et al. 2013). He presented the following argument, which he called the Personality Argument for the Termination Thesis (I refer to it as PATT) (p. 61):

1. When a person dies, he loses the property of being a person.
2. However, when a person loses the property of being a person, he ceases to exist.
3. Therefore, when a person dies, he ceases to exist.

Introducing the following three versions of the arguments, person understood as a biological entity, person (b), person understood as a psychological entity, person (p), and person understood as a moral entity, person (m), he analyzed these three concepts based on three definitions.

Biological:

Dx1: x is a person (b) at t = df. x is a member of the subspecies Homo sapiens sapiens at t.

Psychological:

Dx2: x is a person (p) at t = df. x is able at t to conceive of itself as itself existing before t, and x is able at t to conceive of itself as itself existing after t.

Moral:

Dx3: x is a person (m) at t = df. x has a serious moral right to life at t.

With these definitions (which he arrived at after an extensive analysis of the three concepts), he tests them out against PATT as follows:

1. When a person (b)/(p)/(m) dies, he loses the property of being a person (b)(p)/(m).
2. However, when a person (b)/(p)/(m) loses the property of being a person (b)/(p)/(m), he ceases to exist.
3. Therefore, when a person (b)/(p)/(m) dies, he ceases to exist.

Feldman rejects all three alternatives. The first alternative, the biological, because he sees no reason to hold that premise 1 is true. He thinks one is still a member of one’s species after death. He asserts that if one found a body in a glacier, tested its DNA, and found it to be identical to DNA from *Homo sapiens sapiens*, then one would say that one had found a member of *Homo sapiens sapiens*.

The second, the psychological, he rejects because he holds that one still exists even when one loses the property of being a person (p). A comatose person still exists, he claims.

And the third, the moral, is rejected since it seems to Feldman to be totally unpersuasive to argue, for example, that a person (m) has ceased to exist after having lost his serious right to life because of crimes he has committed.

Feldman’s arguments presuppose that the property of being a person is essential to existence. If he can show that first, people can lose their personality by dying or otherwise, and second, when they lose their personalities, they do not cease to exist, he has proven that PATT is wrong. However, of the three concepts it seems only one, person (b) is an interesting case. To claim that a comatose person or a person waiting for his execution does not exist seems to be a position very few holds. They may not exist as person (p) or person (m), but that is not what we mean when we debate whether death means nonexistence, because we hold the body to be *dead*, not comatose or in a prison.

Feldman argues that dead bodies still exist as persons (b), because to be a person (b) one does not need to be alive. Although he accepts that: “If the property of being alive, for example, were an essential property of the things that have it, then it would follow that something goes out of existence whenever something loses its life.” (Feldman 2000, p. 106). And furthermore, that “…the doctrine that people cease to exist as persons (in any such sense) when they die is relatively uncontroversial. But this view is distinct from TT, 20 for TT is not the view that people cease to exist “as people” when they die. It is the view that they cease to exist *simpliciter* when they die.” (p. 100). This is a bit confusing since Feldman in his argumentation as discussed above, argues that when we are deprived of our personalities, we do not cease to exist. He argues that we can still be persons (b) after death. Very few deny the existence of dead bodies. What I argue is that when I die, the dead body is no longer me – it is a mere thing.

One of Feldman’s arguments for this claim that people do not cease to exist by dying, seems to be his intuitions about what to think of the remains of a human found in a glacier with DNA identical to DNA from Homo sapiens sapiens, as already mentioned (Bradley et al. 2013). Moreover, his intuitions seem to be based on either the claim that life is not a necessary property of horses or *Homo sapiens* or that the loss of an essential property does not make the entity lose its existence. I do not further discuss this last claim since it seems to conflict with the definition of an essential property. However, one must first have an idea of what the thing in question is to be able to identify an essential property. More generally, we must seek the definition of horse or a member of *Homo sapiens* elsewhere.

A possible argument in favor of the idea that a corpse is different from a human being, although with the same DNA, is to refer to Leibniz’s law of the indiscernibility of identicals, as a corpse or a dead horse is undisputedly different from a living human being or a living horse. Therefore, it can be argued that this difference proves that a corpse or a dead horse is no longer identical to a living human being or horse and must thus be something else. However, this does not help us either, because Feldman simply claims that life is not an essential property of a human being or a horse. Thus, although they are different, death has not transformed them into mere things. It seems that our fallback position must simply be that this is a question of semantics. If someone wants to call a corpse or a dead horse a human being or a horse, this is their decision. However, as the time of death moves further into the past (or the corpse is, for example, burnt) and the dead human being or horse decomposes more and more, it presumably becomes increasingly counterintuitive to maintain the position that they are the same as living members of the species in question, although they are dead. At some point, most people would accept that a transformation has taken place. Then why not at death, avoiding a problematic grey-zone later where it could be debated whether the person exists or not. As also Olson argues, although a newly dead may resemble the former living person, it is a very different thing (Olson 2004).

Feldman asked us to accept his argument that he and his friends will not cease to exist when they die. This assertion is based on his claim that he is his body, which normally continues to exist (at least for a while). Therefore, he could say that a person continues to exist as a corpse. He (Feldman) existed as a fetus without any psychological capacities; thus, he asked why he cannot exist as a corpse. Feldman said that the *person (b)* continues to exist as a corpse. This appears to presuppose that there is something essential about people that survives death. Many people believe that they have a soul that survives death. Is this what Feldman was thinking? Probably not, because he claimed that he would exist after death but also admitted that he would then be dead (Feldman 1992). He would probably claim that he was still alive if he thought that he had a living soul after the death of his body. Thus, Feldman seems to identify himself with his body, whether dead or alive.

It seems that some of the discussions on personal identity are semantic. These questions do not appear to be about facts. If they were, what would be their truth makers? Is there some fact in the world that provides the answer to the question of whether a dead body is a mere thing? Surely, this is not an ontological question but rather an ethical and semantic question to which the answer is not found but made. Moreover, an accepted definition of death is the total and irreversible destruction of the brain. Without a functioning brain, the corpse is accepted to be a mere thing that can be buried, burnt, or used to help others by taking its usable organs.

### Conclusion about the Mere thingness of the corpse

As previously argued, I believe that death is the transformation of the person into mere thingness. This is because life is the essential property of a member of the species *Homo sapiens*. Life is also the essential property of being a horse, a dog, or anything else that has a life. (For other things, we probably hold that its purpose is essential. A car that is irrevocably broken and therefore cannot be driven is no longer a car. A broken axe is no longer an axe until it is repaired.)

The definition of death is not the focus of this article. However, as mentioned above, most people accept “brain death” as a definition of death (see Veatch and Ross 2015, pp. 2–5, for a summary of different religious communities’ beliefs about this). Therefore, in the discussion on whether the body or the psychological or moral properties are essential for personhood, most people appear to believe that life is the essential property that is lost when the subject is declared brain dead (although, of course, the brain is not only important for consciousness)—or else, if one held the person still to exist, it would be problematic to take organs for transplantation or to burn or bury the dead, but still existing, body. Veatch and Ross also argued that the transformation caused by death is from a member of the human species to something else, which is *not* a member of the human species.

We do not necessarily have specific names for dead animals. Therefore, we still call a once-alive horse a dead horse, a once-alive dog a dead dog, and so on. However, we do have names for dead humans: corpses or cadavers.^21^

Although there is some discussion on how to explain how and when the dead body came into existence and whether it is numerically identical to the former living human being, it would seem absurd to deny that there is a fundamental difference between the corpse and the former living human being, regardless of semantics. A corpse is essentially different from the living animal, the living person, the living organism, or whatever a person chooses to call them.

## Posthumous harm

The literature on posthumous harm is extensive and focuses on either showing that the antemortem person is harmed by events that occur after his death (i.e., the Feinberg-Pitcher position^22^; see below) or that the dead has or had interests and rights that should be respected. There is a common understanding and agreement that the remains of the dead, the corpse, the ashes, or the buried remains of the body (a mere thing, as I argue) is not an entity (a thing) that can be wronged or harmed. However, the question is who is harmed, when, and how.

This discussion generally focuses on showing that death can be bad, although the dead cannot be harmed. However, my focus in this paper is not on the philosophy of death but rather the conscription of cadaveric organs. However, some arguments from the debate on posthumous harm are relevant, as some scholars argue that the dead still have interests that should be respected (e.g., that their organs should not be taken). Moreover, this position may be defended with reference to the Feinberg-Pitcher position.

Wilkinson addressed the issue of the missing subject: how can anything harm a nonexistent person?^23^ (Wilkinson 2011). He did this by relying on the Feinberg-Pitcher position, which argues that people are either postmortem or antemortem, depending on temporal perspective (Fischer 1993). According to the Feinberg-Pitcher position, antemortem people can be influenced by occurrences that take place after they died. To avoid the problem of backward causation, they both use Cambridge changes and argue that the harm to the antemortem person does not occur when the harmful postmortem event takes place, but that it has been a harm all along. The postmortem occurrence made it true that the person was harmed all along because of what would occur later (after his death). His goals were frustrated if his organs were removed against his will after death. The removal of his organs, however, is not causally linked to the frustration of his goals, but logically, Wilkinson argues, and thereby avoiding the problem of backward causation.

Arguments to the effect that events or acts that occur after death can be harmful include the son who sells his father’s body to a medical school for dissection instead of following his wish to be buried, the Olympic gold medal winner who is falsely accused of foul play after his death and loses his medal, and destroying someone’s reputation after his death. These arguments are based on a certain intuition and understanding of harm: if something happens after death that can be linked to the dead person in some negative way, that person would be harmed not when he is dead but when he was still alive, as the antemortem person whom he once was. One may associate this with Aristotle’s arguments in the *Nicomachean Ethics* that a person’s life cannot safely be called blessed before he is dead: “For to some extent it does seem that something may prove good or bad for someone who is dead, if indeed there are also good or bad things for someone who is living but not actively perceiving them – for example honor and dishonor, and children or descendants generally who do well or who suffer misfortunes” (1100^b^; (Aristotle and Reeve, 2014, pp. 14–15).

The intuitions one may have regarding posthumous harm may differ, as shown by the rather extensive literature on the subject, especially when it comes to unexperienced harms. The lack of experience may either be caused by the subject not knowing or because the subject is missing (i.e., because he is dead). Arguments for harms that will neither be experienced in any way by the subject nor affect them in any way may be a case of semantic differences. People seem to have different understandings of the concept of harm.

The Feinberg-Pitcher position on the badness of death seems to view harm as the thwarting of interests but without affecting the “harmed” subject. The Feinberg-Pitcher position is much discussed and with arguments for and against, with perhaps Palle Yourgrau as the clearest opponent: “Now one would think that mere contemplation of the ontological knots Pitcher has tied himself into would be a sobering inducement to seek an alternative path” (Yourgrau 2000).

What does all of this have to do with organ transplantation? The two parties that are closest to the question of whether to use the corpse’s organs are the antemortem person and the postmortem person’s family. The corpse, I hope that we can agree, has no interests, although some legal rights, e.g., to be treated with respect, and a contingent right relevant for organ procurement, depending on what the antemortem person may have expressed as his will on this, as explained in Sect. 1. It is, of course, problematic to say that these rights are the rights of the corpse, as the corpse, which does not have any interests in the matter, would normally be said to have no rights, as already argued.^24^ Legal regulations presupposing interests and rights with the dead is an example of the somewhat irrational intuitions that motivated these regulations.

## Ownership and a solution to the problem of transfer of ownership

Before concluding this paper, it is necessary to briefly present a few arguments related to the problematic issue of who owns the cadaveric organs and, by extension, can legally transfer their ownership to the recipient. Because I claim that the dead has no interests and rights and that any interests and rights that the family may have or claim to have, are overridden by the much more important interests of patients on waiting lists, the question of ownership must be solved.^25^

One may try to overcome the problem of ownership by instead discussing the right to use. The two concepts, ownership and right to use, are not clearly divided. Since ownership is defined by a bundle of rights and the right to use is typically one of the rights in this bundle, the difference between ownership and right to use is a question of degree, not kind. Therefore, discussion of one’s transplanted kidney as owned by the dead donor, not oneself, seems odd because ownership, in this case, would be no more than the right to use. The donor is dead and therefore has no ownership rights that could be held against the user. Even if the donor was alive, all rights that are typically part of ownership, such as the right to reclaim the thing, the right to destroy it, or the right to give it to another person, are transferred to the receiver of the kidney or, rather, have been relinquished. Thus, one should accept transfer of ownership in this case, which would also seem more natural. However, we must then resolve the question of transfer.

On the one hand, this may seem like a rather abstract question. On the other hand, it seems odd to have implanted organs that belong to someone else, which is nevertheless what is recommended in a Norwegian official report, as we have seen above. How can the body be legitimately donated if it and its parts do not belong to anyone (Quigley 2012)?

Assuming it is correct that the body belongs to the deceased, see Draft Resolution *Transplantation Act and Autopsy Act (*Prop.38 L, p. 37 (*Prop.38 L* (2014–2015) *Transplantasjonslov og obduksjonslov*, 2014)) and the White Paper (NOU 2011: 21 - *When death serves life. A proposal for new laws on transplantation, autopsy and delivery of corpses*, pp. 125–128, then the deceased has the power to transfer its ownership to the recipient. Furthermore, it is argued in Prop. 38 L that the deceased must consent to the donation. This seems odd, as it is also stated that the deceased no longer has any rights or interests. This statement likely means that the antemortem person had ownership of his body. However, the question of transferring its ownership to the receiver is still problematic, even if the antemortem donor had accepted this. How is such a transfer of ownership intended to take place? It cannot occur before the donor has died. It would be strange to say that the body or body parts of the still-living donor belong to someone else. However, after death, the donor has no rights and therefore no ownership of the dead body or its parts and, by extension, no power to transfer ownership to the recipient.

This problem was discussed by Hillary Steiner with regard to the bequest of property (Steiner 2022), but it is perhaps even more important in relation to body parts. Steiner concluded that one must accept the testamentary transfer of ownership as a necessary legal fiction. In our case, however, the situation is both more complex and simpler, depending on what one concludes regarding ownership of the body. If the body belongs to the dead, then a transfer of ownership by the donor seems to be impossible since the dead has no legal right to do such a thing. However, if the body belongs to society or the state, it has become public. Accordingly, society may have the power to donate organs (i.e., transfer ownership) to the recipient. This was also Steiner’s conclusion: “…a correlative duty of title transfer can be owed neither to the bequeather nor to the heir and can, therefore, be owed (if at all) only to the state” (p. 72). For different reasons (the problem of the sensibilities of the family), Harris also advocated for the state as the owner of the cadaver (Harris 1992).

This point, namely that the legal status of the body is important for the transfer of ownership, seems to be overlooked in the literature on organ donation^26^. Most authors appear to focus on the rights, interests, and autonomy of the donor (alive or dead) and thus the importance of consent, without realizing that, if negating the implications of death for the “thingness” status of the corpse, and therefore also the “donor’s” loss of rights (if not legal, then logical), and the state’s role in organ procurement activities, neither voluntary nor the obligatory use of organs where ownership follows the organs, can be defended since transfer of ownership presupposes ownership. Therefore, when Simona Giordano (Giordano 2005) argued that the body is not a republic (i.e., a public property or a res publica), she overlooked that the fiction of the state as a mediator or an interim owner of the dead body is necessary for a logical transfer of ownership.

However, the case of conscription is different. Conscription may be compared to the state’s authority to expropriate property (against compensation). In this case, the transfer of ownership is not questionable; it is expropriated by the state. However, this presupposes that the body or some of its parts are expropriated from the owner. To navigate the philosophical and logical problems of attributing ownership to the corpse, it is possible to adopt a principle of inheritance of the body, as discussed by Teck Chuan Voo and Søren Holm (Voo and Holm 2014). This would overcome the problem of succession. Moreover, as inheritance would be like normal property inheritance, problems of consent, autonomy, interests and rights seem less problematic. The problems could, however, emerge in the next step, when the family has inherited ownership of the dead body. Nevertheless, the state could conscript or, rather, expropriate the body against compensation (e.g., by covering costs for the funeral). As the new owner, the state could then donate the body for transplantation.

## Conclusion

I argued that the conscription of cadaveric organs for transplantation is preferable to the present system in Norway and to systems in every other country in which organs that could have been used to save lives and improve well-being are instead burnt or buried. I presented well-known ethical theories to support my argument. Furthermore, I posited that counterarguments fail because they are based on flawed argumentation about the interests and rights of the dead. Arguments against conscription that are based on a feeling of distress that the antemortem person or the family may experience are dismissed as much less important than saving lives and improving the well-being of potential organ recipients and their families. I argued that death transforms the living person into a mere thing, albeit a valuable thing for others. However, the value of the corpse is neither moral nor intrinsic. It is a relational value, a value based on what value the corpse has for others, most importantly, the value for people in need of new organs, but also the value it has for family and friends, as a symbol of what the person once was for them. However, the use of organs for transplantation should not influence the family’s feelings in any negative way but, if anything, be a comfort in their sorrow.

Accepting routine taking of organs from suitable donors would shorten waiting lists, and therefore save lives, and increase well-being and happiness. It would also save health personnel from the burden of asking for consent from a family in grief, and also save some families from a difficult decision in a very difficult situation. I further think that such procedures would be accepted, at least after some time, by society at large, the way forensic autopsies are accepted.

## Footnotes

1 The argument for the thingness of the corpse is inspired by Søren Holm in private conversation.

2 In Norway, 72% of potential donors accepted donations in 2022 (Accept given by the dead before death, or by the family after death). However, there were 405 people on waiting lists as of December 31, 2022, and 24 died while waiting for an organ transplant. In the United States, 17 patients died on waiting lists per day in 2021 (Pemberton 2023), and only around 55% of the population was willing to grant formal permission to become a donor (Taylor 2005 p. 9).

3 Semantically, “conscription” may imply the taking of organs against the “donor’s” will. However, in the case of cadaveric organs, there is no such will since the “donor” no longer exists. In this paper, conscription is understood as the taking of organs regardless of the wish of the antemortem person (and the family).

4 “Cadaver” is a technical notion for a dead person (i.e., a dead body). In this context, it does not refer to a dead animal.

6 I took inspiration on this issue from Bjørn Hoffman, Søren Holm, Jan Helge Solbakk (Solbakk et al. 2009; Hofmann et al. 2006a, b, c), George Lakoff, and Mark Johnson (Lakoff and Johnson 2003).

7 That this utilitarian argument can be used for a policy to also take organs also from the living was maintained by, for example, Cecile Fabre (Fabre 2006b) and John Harris (Harris 1975).

8 Which, however, some deny. See more on this in Chap. 4.

9 This is the Epicurean argument that death is neither good nor bad.

10 I thank Søren Holm for this reference.

11 The strength of these feelings is illustrated in a letter to the editor in the Danish newspaper *Weekendavisen* from February 23, 2024 (No. 8), in which a reader comments on a discussion to implement an opt-out system for organ donation in Denmark. He argues that pictures of Nazi concentration camps and Russian mass graves in Ukraine are examples of a brutalized view of the bodies of dead human beings and therefore also human beings in general. Furthermore, he asks if it is along this horrible path that the government wants to go with its possible acceptance of such improper treatment of corpses., which would be the case if organs are taken without explicit consent.

13 The free-rider problem refers generally to the case that some people take advantage and leaving the burden to others. Transplantation is an example in which people may be unwilling to be donors but still want to be organ recipients if they are in need. Conscription would eliminate this problem. However, allowing a few people to be recipients without also being potential donors could endanger the acceptance of implementing a system of conscription. However, as the “opt-out” possibility would be very limited, to maybe only some orthodox Jews and some Mormons, this problem would probably be reduced to very few, if any, in Norway. Thus, although it is of philosophical interest, the principle of reciprocity or the “free rider” problem is in itself a major topic in philosophy (Rawls 1958, 1973; Nozick 2012; Arneson 1982; Klosko 1994) it cannot be covered in this article.

14 There may be cases where stolen goods are given, which may produce the same feelings. A discussion of such cases will, however, bring the discussion in this article out of focus.

15 J. R. Karlsen et al. argued that human biological material should perhaps be understood as “no-things,” as we lack a suitable third category that is not person and not thing (Karlsen et al. 2006). See also Daniel Sperling’s concept of the Human Subject, as mentioned above.

16 Although some may argue that Epicurus’s hedonism, by definition, requires an experiencing subject, which is not necessarily the case for other theories, I claim that, for example, objective list or desire theories also necessitate an experiencing subject. Therefore, regardless of one’s ethical (secular) viewpoint, nothing matters anymore after death.

17 Divine Ndonbi Banyubala showed that this can be significantly different in other cultures in the abovementioned article.

18 The nonvalue of death is understood as the Epicurean claim that “death is nothing to us.” In other words, it is neither good nor bad.

19 This also leads to a situation in which the feelings and arguments of family and friends have relatively higher importance than any consideration of the deceased person’s antemortem thoughts on the matter. I thank Carl Tollef Solberg, who highlighted this to me in private conversation.

20 The Termination Thesis.

21 However, some deny the existence of corpses, as Hershenov points out, referring to Olson and van Inwagen: “What is called a ‘corpse’ is a *mere* plurality of things that no more compose a genuine individual than do my hat, jacket and backpack when they lie in contact with one another on my coffee table” (author’s emphasis) (Hershenov 2005, p. 40).

22 Joel Feinberg, Harm to Others, and George Pitcher, The Misfortunes of the Dead (Fischer 1993).

23 A nonexisting person is an oxymoron, but I think that it is understandable.

24 However, Sperling seems to see this differently (see above).

25 That this is a complex and difficult issue, is shown by Sperling’s comprehensive description of the different questions arising.

26 But not by Daniel Sperling which, as already mentioned has treated this and most of the connected issues in his book Posthumous Interests.
